# Parent perceptions regarding virtual pediatric dental clinics during COVID-19 pandemic: a cross-sectional study

**DOI:** 10.7717/peerj.15289

**Published:** 2023-08-14

**Authors:** Sara Ayid Alghamdi

**Affiliations:** Department of Preventive Science, College of Dentistry, Majmaah University, Al-Majmaah, Saudi Arabia

**Keywords:** COVID-19, Pediatric dentistry, Telehealth, Teledentistry, Children

## Abstract

**Objective:**

To assess the attitudes and responses of parents of pediatric patients towards virtual dental clinics during COVID-19.

**Material and Method:**

A total of 102 parents of pediatric patients who were scheduled for virtual (video or telephonic) clinic appointments for new patient consultations and follow-up clinics were included in the study. Parents and patients could attend the virtual clinic from a personal computer, tablet, or smartphone. An electronic self-administered questionnaire was sent to the parents through email after consultation (video or telephonic) which consisted of demographic data and a ten-item, five-point Likert-scale assessing: (i) parent satisfaction; (ii) ease of use; (iii) the effectiveness including increasing access to clinical services; (iv) reliability of the teledentistry system and (v) usefulness for patients. Statistical analyses used were *t*-test, one-way ANOVA test, Shapiro–Wilk and histogram.

**Result:**

Out of 102 parents, 52 attended video clinics and 50 attended the telephonic clinic. The majority of the parents were between 30–39 years of age, and about 73% had no previous experience with either virtual or telephonic consultation. Ninety-four percent of parents were satisfied with vide clinic consultation, and most of them agreed that accessing clinical services through a video clinic was easy to understand, comfortable, and time-saving. The majority of the parents (94%) agreed and strongly agreed that they will use video clinics again in the future for consultation.

**Conclusion:**

Parents’ response to the use of virtual clinics for pediatric dentistry during COVID was positive. The majority of the parents stated that they would consider using teledentistry for future consultation.

## Introduction

Dentistry has seen many ups and downs during the past two years since the emergence of coronavirus disease (COVID) (Wajeeh et al., 2021). Direct face-to-face consultations between patients and dentists were prohibited for a considerable period of time except for any emergency treatment, which prohibited patients from receiving routine dental care during the pandemic (Wajeeh et al., 2021; Quinn et al., 2020). This has led to substantial technological innovations in an attempt to continue providing oral health care to patients (Quinn et al., 2020; Jampani et al., 2011). One such crucial innovation is teledentistry, which helps to continue dental practice without increasing the risk of community virus transmission. Teledentistry, as the name suggests, combines telecommunication and dentistry (Yoshinaga, 2001). It refers to the exchange of clinical images and information over remote distances for oral health consultation and treatment planning (Singhal, Mohapatra & Quiñonez, 2022; Cagetti et al., 2021). It involves the use of computers, laptops, tablets, smartphones, and the internet as means of communication between patients and clinicians (Kumar et al., 2021; Deshpande et al., 2021). Teledentistry allows dental clinicians to provide consultation, make the provisional diagnosis, treatment planning, prescribe medications, advise referrals and educate patients about the importance of oral health care without actually meeting the patient in person (Deshpande et al., 2021; Santana et al., 2020; Nassani et al., 2021). The major advantage of teledentistry is that it permits minimum attendance in clinical settings thus reducing the risk of COVID transmission (Aldhuwayhi et al., 2021). This is of paramount importance, particularly for the clinician and patient protection. Teledentistry includes both virtual/video clinics or telephonic consultation (Consolo et al., 2020). It allows face-to-face consultation between patient and clinician when utilizing video clinic appointments, which saves cost and time of traveling and vehicle parking and waiting time (Nassani et al., 2021). In addition to this, video consultation allows clinicians to observe and communicate with their patients without any physical contact, thus reducing the anxiety and fear of virus infection, especially among immunocompromised patients who are at a higher risk of viral infection (Goriuc et al., 2022). Teledentistry has proved its usefulness in various disciplines of dentistry including oral medicine, orthodontics, endodontics, community dentistry, periodontics, and pediatric dentistry with satisfactory outcomes (Deshpande et al., 2021; Santana et al., 2020; Nassani et al., 2021; Menhadji et al., 2021; Ben-Omran et al., 2021; Flores et al., 2020; Al-Shammery et al., 2021; Palmer et al., 2005; Ibraheim, Sanalla & Eyeson, 2021; Murthy et al., 2021; Wallace et al., 2021). The purpose of the present study was to identify the attitudes and responses of parents of pediatric patients towards virtual dental clinics and telephonic clinics (teledentistry) and to identify potential ways to improve this experience and make it more applicable in the future among the parents of patients attending the pediatric dental clinic.

## Material and Methods

### Ethical clearance

The present study protocol was approved by the institutional ethical committee at Majmaah University Saudi Arabia, with the number IRB No: MUREC-NOV-11/Com-2021/11-2. Written informed consent was obtained from each participant prior to their inclusion in the study.

### Study sample and size

Parents of pediatric patients attending the virtual and telephonic dental clinic were selected from the patient list in the dental clinics using a simple random sampling method from the dental clinic system of Riyadh. The sample size was calculated using. Parents of patients attended the pediatric dental department, College of dentistry at Majmaah University, Saudi Arabia, during the pandemic situation, they agreed to participate in a virtual pediatric dental clinic consultation (video clinic or telephonic clinic) and gave their written informed consent to participate in the post-consultation online survey included in the study. Parents of either gender who agreed to attend a virtual pediatric dental clinic (video clinic or telephonic clinic) and gave their written informed consent to participate in the post-consultation online survey were included in the study, while those who were unable to give informed consent or who disagree to participate in the post-consultation survey were excluded from the study.

### Data collection

Data was obtained from parents of pediatric patients who were scheduled for virtual (video or telephonic) clinic appointments for new patient consultations and follow-ups over four clinics. Parents were free to choose among the two types of virtual clinics. Patients and parents were allowed to attend the virtual clinic from either their personal computer, tablet, or smartphone. A total of 102 parents participated in the post-consultation survey. An electronic self-structure questionnaire was sent to the parents through email after a virtual consultation. The questionnaire was designed to collect demographic data including age, gender, occupation, and previous telehealth experience. The later part of the questionnaire consisted of a ten-item, five-point Likert-scale questionnaire assessing: (i) patient satisfaction; (ii) ease of use; (iii) the effectiveness including increasing access to clinical services; (iv) reliability of the teledentistry system, and (v) usefulness for patients. The commencement of the study and data collection happened at the end of the year 2021. The video and telephonic clinics were conducted during the month of November 2021. The same person was involved in the consultation for both the clinics and also in data collection. The time commitment for each patient was 15–20 min.

### Statistical analysis

The number and percentage were reported for the age category, gender, occupation, and previous experience with telehealth overall and clinic-wise. The bar chart displayed the previous experience with telehealth overall and clinic-wise. A one-way ANOVA test was used to compare the average total score between age groups and occupations in the telephonic clinic and virtual clinic separately. The *t*-test was used to compare the average total score between gender by the telephonic clinic and virtual clinic separately. The stacked bar charts were displayed to show the telephone and virtual clinic survey results. All analyses were done using Statistical Package for Social Sciences (SPSS) software Version 21.0 (Armonk, NY: IBM Corp).

## Results

**Figure 1:**
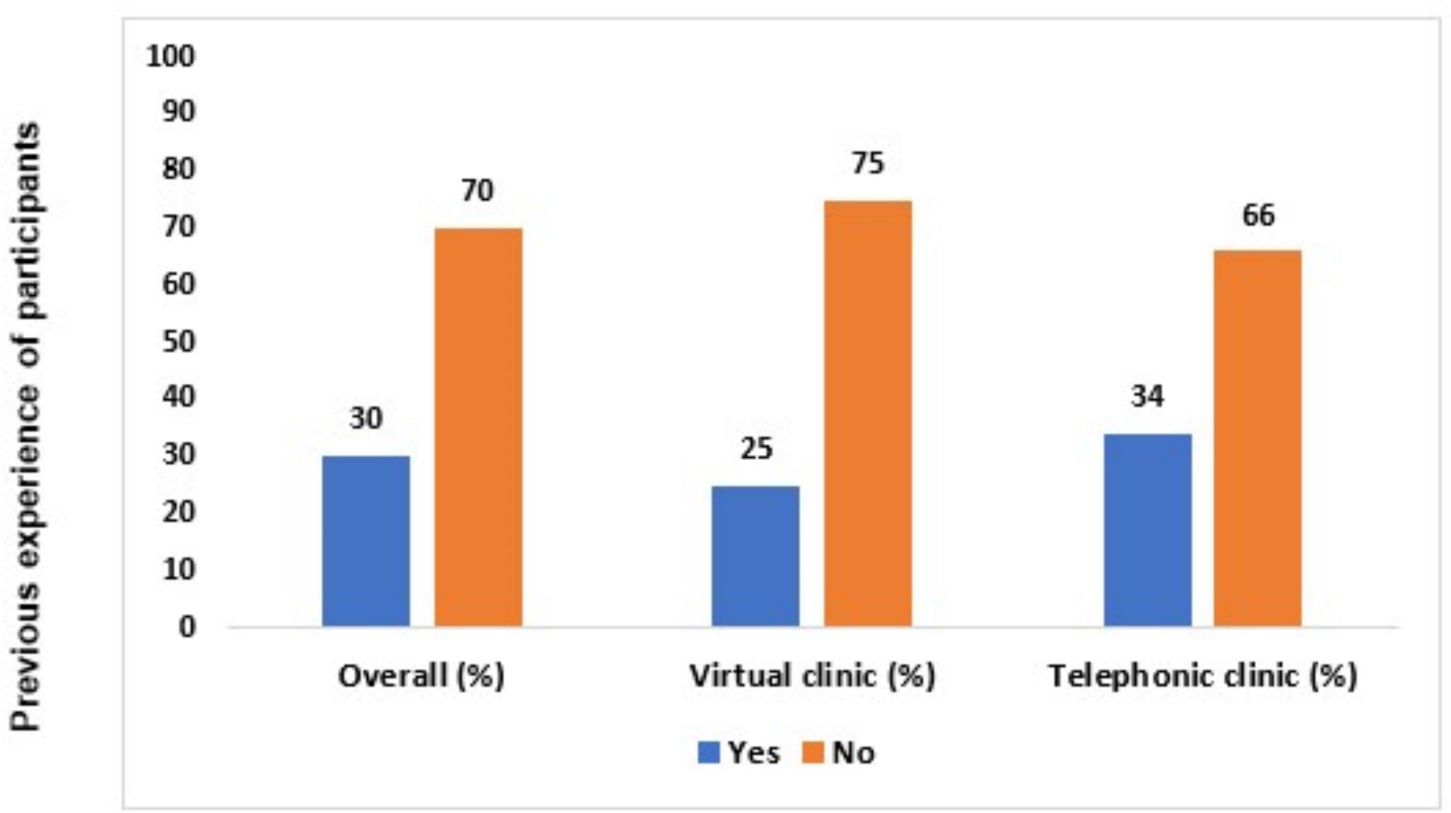
Previous experience of the video clinic and telephonic clinic among participants.

All the parents responded positively to the post-consultation online survey without any complaints regarding their understanding of the questionnaire. Out of 102 parents, 52 parents opted for video clinics while 50 chose telephonic clinics for consultation. In the present study, 63.7% of the parents were female while 36.3% were male and the age range of parents was <19–60 years, with the maximum number (41.2%) of parents between 30–39 years. Out of 50 (49%) employed parents, 23 attended video clinics while 27 chose telephonic clinics. However, out of the 17 (16.7%) parents who were students, 14 opted for video clinics and three attended telephonic clinics (Table 1). Approximately 70% of the parents had no previous experience with either type of telehealth communication, among them only 25% of them had any experience with virtual clinics while 34% of them had any experience with a telephonic clinic (Fig. 1).

None of the comparison of overall means scores for various age groups, gender, and occupation that participated in the study (Table 2) was found with statistical significance for the telephonic clinics (*p* > 0.05) and video clinics (*p* > 0.05). Among the age groups, the parents belonging to the <19 years group reported minimal mean scores (13.2 ± 7.15), while parents of the 50–59 years age group achieved high mean scores (19 ± 3.56) for telephonic clinics. The males observed with higher mean scores for telephonic clinics than video clinics (*P* > 0.05). The females observed higher mean scores for telephonic clinics than video clinics (*p* < 0.05). In the case of telephonic and video clinics, males had lower mean scores than females (*p* > 0.05). Among the age groups, the parents who were students achieved high mean scores (22.67 ± 3.05), while parents of the employee group achieved low mean scores (16.63 ± 4.01) for telephonic clinics. Within the age groups, the parents of 30–39 years had higher mean scores for telephonic clinics than the video clinic, which was statistically significant (*p* < 0.001). The parents who students and employees had high scores for telephonic clinics(22.67 ± 3.05 and 16.63 ± 4.01) compared to video clinics(10.14 ± 0.53 and 11.30 ± 3.65) with statistically significant (*p* < 0.001).

**Table 1:** Description of demographic and professional characteristics of study participants.

**Characteristics**	**Overall *n* (%)**	**Video clinic *n* (%)**	**Telephonic clinic *n* (%)**
Age (years)			
<19	15 (14.7)	10 (19.2)	5 (10.0)
20–29	12 (11.8)	06 (11.5)	6 (12.0)
30–39	42 (41.2)	20 (38.5)	22 (44.0)
40–49	24 (23.5)	11 (21.2)	13 (26.0)
50–59	9 (8.8)	05 (9.6)	5 (8.0)
Gender			
Male	37 (36.3)	21 (40.4)	16 (32.0)
Female	65 (63.7)	31 (59.6)	34 (68.0)
Occupation			
Student	17 (16.7)	14 (26.9)	3 (6.0)
Unemployed	28 (27.5)	12 (23.1)	16 (32.0)
Retired	07 (6.9)	03 (5.8)	4 (8.0)
Employee	50 (49.0)	23 (44.2)	27 (54.0)

**Table 2:** Comparison of demographic characteristics on the total scores.

**Details**	**Telephonic clinic**		**Video clinic**		**(Comparison between telephonic and video clinics)** ***P* value**
	**Mean (SD)**	***p*-value**	**Mean (SD)**	***p*-value**	
Age (years)					
<19	13.20 (7.15)	0.302	10.20 (0.63)	0.414	0.194
20–29	16.50 (3.88)				13.50 (4.81)		0.262
30–39	17.24 (4.24)				12.40 (5.67)		0.003
40–49	19.00 (3.42)				10.55 (1.29)		<0.001
50–59	19.00 (3.56)		12.00 (2.82)		0.013
Gender					
Male	16.88 (3.20)	0.732	11.62 (4.58)	0.938	0.0004
Female	17.35 (5.08)		11.71 (3.77)		<0.001
Occupation					
Student (Parent)	22.67 (3.05)	0.129	10.14 (0.53)	0.120	<0.001
Unemployed	16.69 (5.27)				13.75 (6.29)		0.193
Retired	19.00 (3.56)				13.33 (3.05)		0.078
Employee	16.63 (4.01)		11.30 (3.65)		<0.001

**Figure 2:**
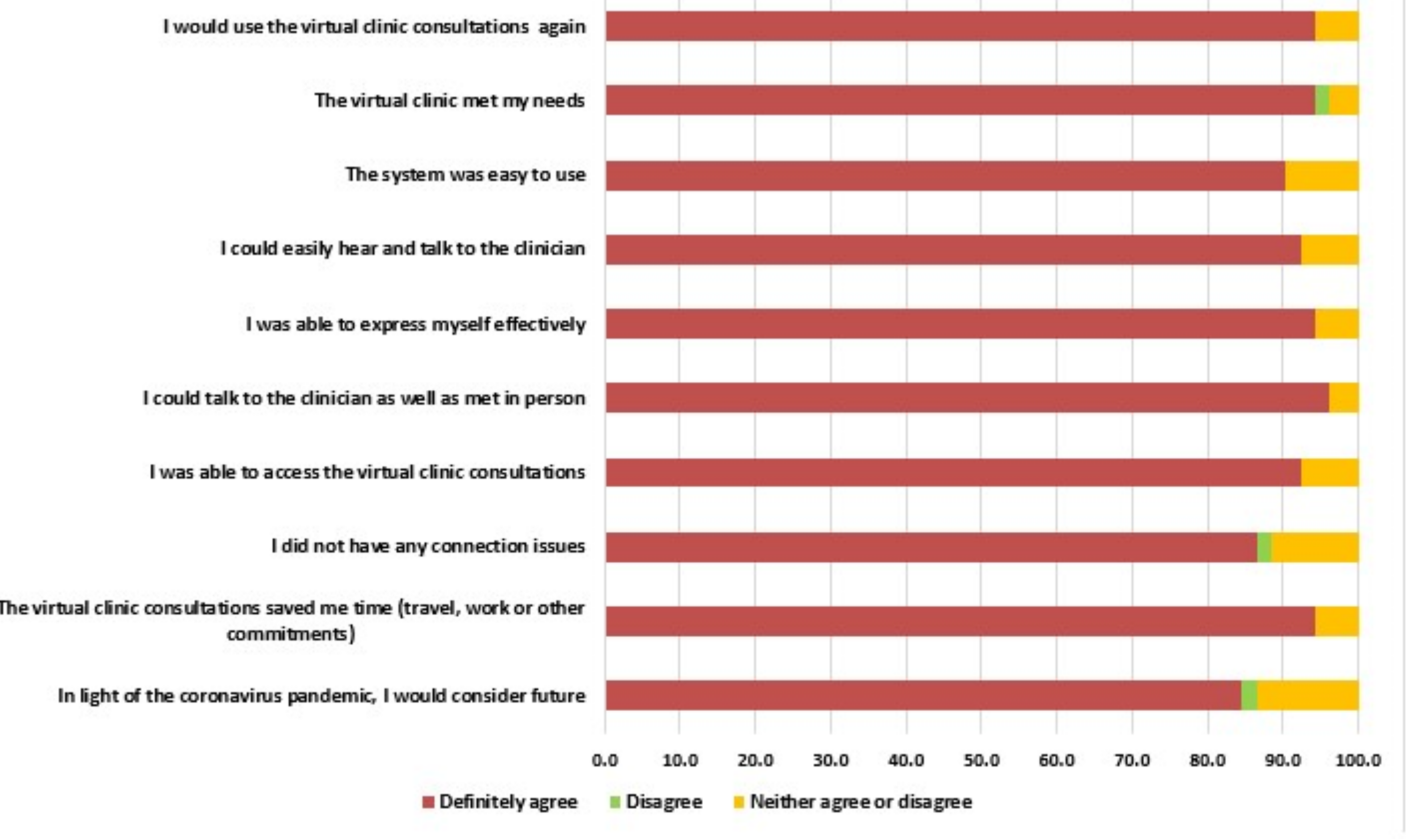
Details of video clinic survey among the participants.

**Figure 3:**
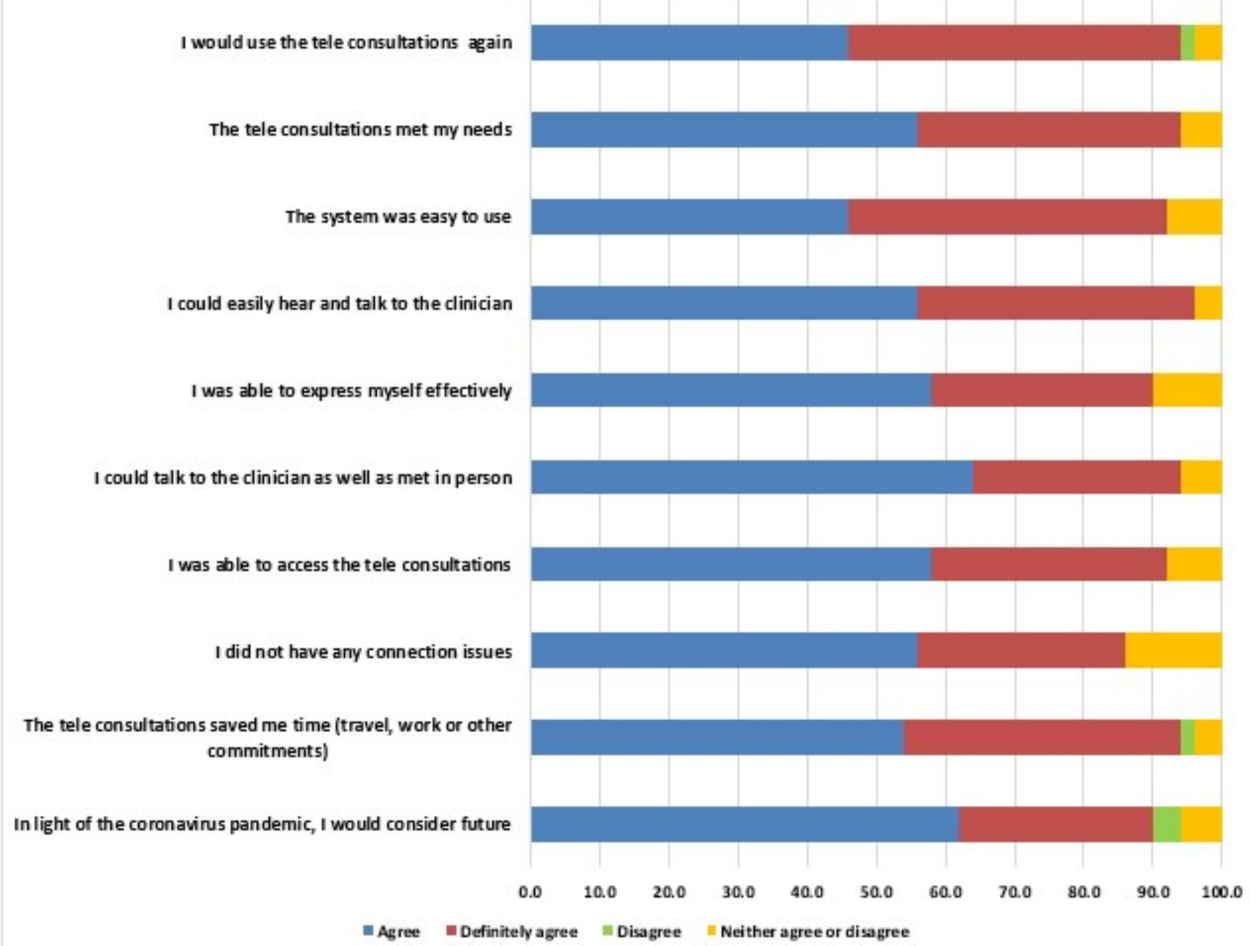
Details of telephonic clinic survey among the participants.

Almost 87.3% of parents stated that they would consider video clinics for future consultations amid the coronavirus situation. About 94% of parents were satisfied and happy with video clinic consultations since they saved their time (Fig. 2). Most of the parents (89%) were able to access video clinics through their personal computers, tablets, and smartphones without facing any difficulties, out of which 61% strongly agreed that accessing clinical services through the virtual clinic is not complicated. However, one parent has faced connection issues during virtual consultation. The majority of the parents (64%) strongly agreed that they were able to talk and express their concerns to the clinician and felt like meeting the clinician in person through the virtual clinic. However, 5% couldn’t agree or disagree. Approximately 94% of parents agreed and strongly agreed that they were able to hear, understand and communicate with the clinician, while 6% neither agree nor disagree. 91.2% of parents found the electronic system of virtual consultation easy to understand and use. The majority of the parents (94.1%) agreed and definitely agreed that virtual clinic consultation has fulfilled their needs and answered their queries and concerns while 1% of parents disagreed. Approximately 94% of parents agreed and strongly agreed that they will use virtual clinics again in the future, while 5% neither agreed nor disagreed and 1% disagreed to use teledentistry in the future. The responses of participants on virtual dental clinics were illustrated in Fig. 2 and telephonic clinic were illustrated in Fig. 3.

## Discussion

The dental practice has been primarily affected by coronavirus disease worldwide. Since dentistry involves direct face-to-face contact with the patient and many dental procedures involve aerosol production, the dental practice was put to a halt, except for emergency services, for a certain period during the initial phase of coronavirus spread in an attempt to prevent community viral transmission (Mallineni et al., 2022a; Mallineni et al., 2021). This was when the concept of teledentistry caught the attention and has dramatically emerged as a means to continue dental practice without increasing the risk of viral transmission (Mallineni, Bhumireddy & Nuvvula, 2021; Wolf et al., 2021). Although the concept of teledentistry is not new, it is significant and clinical applications were highlighted during the COVID pandemic. Teledentistry was first defined by Cook (1997) as the utilization of video-conferencing technologies for diagnosis and treatment planning over a remote distance (Cook, 1997; Fricton & Chen, 2009). Teledentistry, a subset of telemedicine, has been advocated and encouraged by many hospitals, clinics, and healthcare providers as a means of the preliminary stage of care through telephonic consultation or video consultation, thus maintaining social distancing protocol during COVID (Kumar et al., 2021; Menhadji et al., 2021). It can be considered an adjunct to routine face-to-face dental consultations, especially for pediatric dental care, which may result in better patient management. According to a recent review, it has been suggested that teledentistry could help reduce the demand-supply gap between patients and pediatric dental specialists in areas with limited healthcare facilities. Thus, it ensures safety for both patient and clinician while delivering dental care to patients in need (Sharma, Suprabha & Rao, 2021).

A study from Saudi Arabia assessed mothers’ knowledge of COVID 19 and evaluated their attitude and fear regarding dental visits during pandemics (Farsi & Farsi, 2021). They observed that mothers of children and adolescents adjudged dental clinics as a risky setting for contracting the coronavirus. Thus, they could not take their children to the dentist except in an emergency (Farsi & Farsi, 2021). The importance of teledentistry in dental operatory was discussed among dental undergraduate students (Almulhim et al., 2021) and the general population (Alassaf et al., 2021). To our knowledge, this is the first study that evaluated the usefulness of teledentistry from the parents’ pediatric patients’ perspectives. The results of the present study showed that the majority of parents were satisfied with using teledentistry in all five domains of patient satisfaction, ease of use, and effectiveness, including increasing access to clinical services, reliability of teledentistry system, and usefulness for the patients. Parents confirmed that the teledentistry technology facilitated easy access to a pediatric specialist and reduced travel time and cost. It also reduces unnecessary referrals and long waiting hours for specialist consultation at hospitals or dental clinics (Mandall et al., 2005; Mariño et al., 2015). Despite the initial reluctance and extra expense, teledentistry has been proven to help minimize the inequalities in oral health (Scuffham & Steed, 2002).  Menhadji et al. (2021) conducted a study to investigate dentists’ and patients’ attitudes toward teledentistry (dental video consultations) and observed that the majority of the patients were highly satisfied after using teledentistry. Similarly, the majority of dentists have found teledentistry very helpful and easy to conduct. More than 70% of the patients strongly agreed that accessing the video consultation from their home was comfortable, ran smoothly, and saved time and the cost of travel. Almost 80% of patients confirmed that they would recommend video consultation in the future. Another study assessed patients’ and clinicians’ satisfaction with virtual consultations for their orthodontic appointment. As stated by the clinicians, a virtual appointment was found to be appropriate in 90% of the cases. 76% of patients admitted that virtual consultation was more convenient when compared to face-to-face consultation and 66% reported that they would prefer virtual consultation, if required, in the future (Byrne & Watkinson, 2021). Parker and Chia observed similar observations in their study (Parker & Chia, 2021).

A similar survey study was undertaken by Rahman, Nathwani & Kandiah (2020) to evaluate patients’ experience after using teledentistry during the coronavirus disease pandemic. They obtained a one hundred percent response rate with 52 completed surveys over seven clinics, while in the present study, 102 parents completed the surveys over four clinics. They observed 97% and 94% patient satisfaction after using video clinics and telephonic clinics, respectively. These results follow the results of the present study. In their study, 96% of patients stated that they would use teledentistry in the future, especially amid the COVID-19 situation. Similar results were obtained in the present study, where about 94% of the parents confirmed that they would consider teledentistry for future consultation. Another essential advantage of teledentistry that has been appreciated during the COVID situation is that it minimizes unnecessary exposure of patients to coronavirus directly or through asymptomatic carriers, for example, healthcare workers, if they visit the hospital physically (Rockwell & Gilroy, 2020). Densely populated areas have been reported to be more susceptible to virus transmission  (Nuvvula & Mallineni, 2021; Khairat et al., 2020). Wallace et al. (2021) and Ben-Omran et al. (2021) conducted a study to evaluate the potential role of teledentistry in pediatric dentistry. They found that routine face-to-face consultations can be reduced to almost one-third by implying a preliminary telephonic consultation for initial appointments. Acceptance of video and telephonic consultations by patients during the pandemic has helped diminish unessential hospital visits and overpopulation of the emergency area. This has decreased the risk of coronavirus disease transmission and ultimately prevented overload on the healthcare providers and healthcare system (Cochrane Library, 2020; Bhumireddy, Mallineni & Nuvvula, 2021). Although virtual clinic appears promising as an aid for preliminary patient consultation during this COVID situation, it is often difficult for older people, those who belong to lower socioeconomic groups or live in remote/backward areas with a lack of internet facility, and those with learning needs to access virtual consultation (Bhumireddy, Mallineni & Nuvvula, 2021; Farronato et al., 2020; Mallineni et al., 2022b). For such patients, telephonic consultation is the best way to provide primary health care during the current challenging situation. The study sample size is around 102 even though the sample size is significantly less, and comparatively, Rahman, Nathwani & Kandiah (2020) study used only 52 responses for evaluation. This sample may be due to confusion among parents due to the COVID-19 pandemic situation. Various researchers (Torres-Pereira et al., 2013; Estai, Kruger & Tennant, 2017; Gomes et al., 2017; Sharma et al., 2020; Steinmeier et al., 2020; Tiwari et al., 2022) have performed studies around the globe to determine the effectiveness of teledentistry in daily dental practice, specifically in oral screening in communal areas. Lin et al. (2022) opined there is a lot of scope in teledentistry to explore. An Indonesian study (Soegyanto et al., 2022) with 654 dentists also reported that teledentistry should benefit clinicians and patients. The findings are not compared with the present study because the current study involved parents of children attending the pediatric dental clinic. Approximately parents could not access the internet and had poor network issues. This was not evaluated in the study, which is a limitation of the study. The study population was parents of a patient pool from a teaching hospital. Therefore, selection bias is warranted. The questionnaire used in the study was a self-administered questionnaire. The data was not analyzed based on the study participant age group distribution and occupation; this was also a potential limitation of the study. However, the results showed interesting observations among Arabian parents, which could be used as a reference for upcoming research.

## Conclusions

Within the limitations, the present study reveals the positive response of the parents of pediatric patients after using virtual pediatric dental clinics and their willingness to utilize teledentistry for future consultations amid COVID-19. Teledentistry has been accepted by patients and their parents in means of video and telephonic clinics due to its easy accessibility, comfort, risk-free and time-saving nature.

##  Supplemental Information

10.7717/peerj.15289/supp-1 Supplemental Information 1 Raw data

10.7717/peerj.15289/supp-2 Supplemental Information 2 Study QuestionnaIre

10.7717/peerj.15289/supp-3 Supplemental Information 3 Data analysis

## Additional Information and Declarations

### Data Availability

The following information was supplied regarding data availability: The raw data is available in the Supplemental File.
