# Supplementary material for: Parent perceptions regarding virtual pediatric dental clinics during COVID-19 pandemic: a cross-sectional study
Source: PeerJ. 2023 Aug 14;11:e15289. doi: 10.7717/peerj.15289 (PMC10434104; doi:10.7717/peerj.15289)
Supplement: Supplemental Information 2 [file peerj-11-15289-s002.docx]

| **Virtual clinic** | |
| --- | --- |
|  | Gender: male/ female |
|  | Age: >20/20-29/ 30-39/40-49/50-59 |
|  | Occupation: Student / Unemployed/ Retired/ Employee |
|  | Previous experience with telehealth: yes/No |
|  | In light of the coronavirus pandemic, I would consider future consultations via virtual clinic |
|  | 1. Strongly disagree b. Disagree c. Agree or disagree d. Agree e. Strongly agree |
|  | The virtual clinic saved me time (travel, work or other commitments) |
|  | 1. Strongly disagree b. Disagree c. Agree or disagree d. Agree e. Strongly agree |
|  | I was able to access the virtual clinic |
|  | 1. Strongly disagree b. Disagree c. Agree or disagree d. Agree e. Strongly agree |
|  | I did not have any connection issues] |
|  | 1. Strongly disagree b. Disagree c. Agree or disagree d. Agree e. Strongly agree |
|  | I could talk to the clinician as well as met in person] |
|  | 1. Strongly disagree b. Disagree c. Agree or disagree d. Agree e. Strongly agree |
|  | I was able to express myself effectively |
|  | 1. Strongly disagree b. Disagree c. Agree or disagree d. Agree e. Strongly agree |
|  | I could easily hear and talk to the clinician |
|  | 1. Strongly disagree b. Disagree c. Agree or disagree d. Agree e. Strongly agree |
|  | The system was easy to use |
|  | 1. Strongly disagree b. Disagree c. Agree or disagree d. Agree e. Strongly agree |
|  | The virtual clinic met my needs |
|  | 1. Strongly disagree b. Disagree c. Agree or disagree d. Agree e. Strongly agree |
|  | I would use the virtual clinic again |
|  | 1. Strongly disagree b. Disagree c. Agree or disagree d. Agree e. Strongly agree |

**Questionnaire**

| **Tele clinic** | |
| --- | --- |
|  | Gender: male/ female |
|  | Age: >20/20-29/ 30-39/40-49/50-59 |
|  | Occupation: Student / Unemployed/ Retired/ Employee |
|  | In light of the coronavirus pandemic, I would consider future consultations via telephone |
|  | a.Strongly disagree b. Disagree c. Agree or disagree d. Agree e. Strongly agree |
|  | The Telephone consultations saved me time (travel, work or other commitments) |
|  | 1. Strongly disagree b. Disagree c. Agree or disagree d. Agree e. Strongly agree |
|  | I did not have any connection issues] |
|  | 1. Strongly disagree b. Disagree c. Agree or disagree d. Agree e. Strongly agree |
|  | I was able to access the Telephone consultations |
|  | 1. Strongly disagree b. Disagree c. Agree or disagree d. Agree e. Strongly agree |
|  | I could talk to the clinician as well as met in person |
|  | 1. Strongly disagree b. Disagree c. Agree or disagree d. Agree e. Strongly agree |
|  | I was able to express myself effectively |
|  | 1. Strongly disagree b. Disagree c. Agree or disagree d. Agree e. Strongly agree |
|  | I could easily hear and talk to the clinician |
|  | 1. Strongly disagree b. Disagree c. Agree or disagree d. Agree e. Strongly agree |
|  | The system was easy to use |
|  | 1. Strongly disagree b. Disagree c. Agree or disagree d. Agree e. Strongly agree |
|  | The Telephone consultations met my needs |
|  | 1. Strongly disagree b. Disagree c. Agree or disagree d. Agree e. Strongly agree |
|  | I would use the Telephone consultations again |
|  | 1. Strongly disagree b. Disagree c. Agree or disagree d. Agree e. Strongly agree |
