# Supplementary material for: Parent perceptions regarding virtual pediatric dental clinics during COVID-19 pandemic: a cross-sectional study
Source: PeerJ. 2023 Aug 14;11:e15289. doi: 10.7717/peerj.15289 (PMC10434104; doi:10.7717/peerj.15289)
Supplement: Supplemental Information 3 [file peerj-11-15289-s003.docx]

Table 1: Description of demographic and professional characteristics of study participant

| **Characteristics** | **Overall (n=102)**  **n (%)** | **Virtual clinic (n=52)**  **n (%)** | **Telephonic clinic (n=50)**  **n (%)** |
| --- | --- | --- | --- |
| Age (years)  >20  20-29  30-39  40-49  50-59 | 15(14.7)  12 (11.8)  41 (40.2)  24 (23.5)  09 (8.8) | 10 (19.2)  06 (11.5)  20 (38.5)  11 (21.2)  05 (9.6) | 5 (10.0)  6 (12.0)  21 (42.0)  13 (26.0)  4 (8.0) |
| Gender  Male  Female | 37 (36.3)  65 (63.7) | 21 (40.4)  31 (59.6) | 16 (32.0)  34 (68.0) |
| Occupation  Student (Parent)  Unemployed  Retired  Employee | 17 (16.7)  28 (27.5)  07 (6.9)  50 (49.0) | 14 (26.9)  12 (23.1)  03 (5.8)  23 (44.2) | 3 (6.0)  16 (32.0)  4 (8.0)  27 (54.0) |
| Previous experience with telehealth?  Yes  No | 30 (29.4)  72 (70.6) | 13 (25.0)  39 (75.0) | 17 (34.0)  33 (66.0) |

Figure 1:


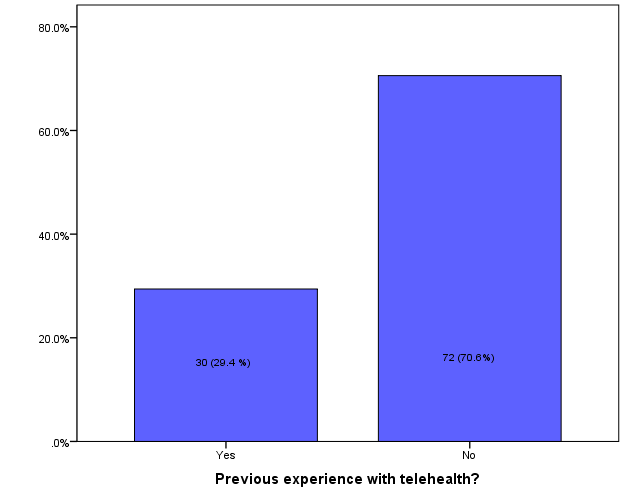


Figure 2:


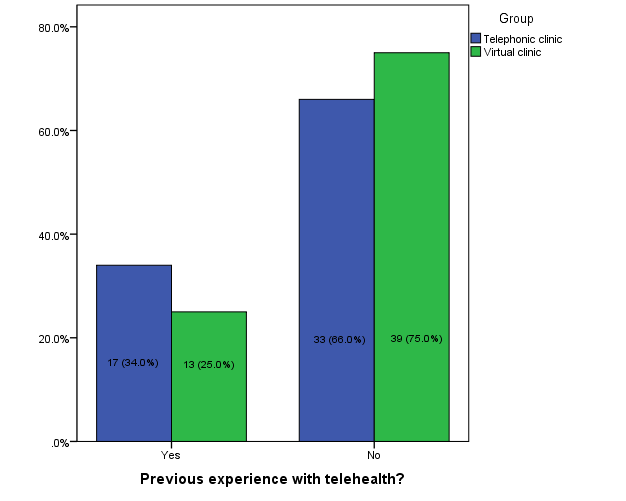


Table 2: Comparison of demographic characteristic on the total score

|  | **Telephonic clinic** | | **Virtual clinic** | |
| --- | --- | --- | --- | --- |
|  | **Mean (SD)** | **p-value** | **Mean (SD)** | **p-value** |
| Age  8-15  16-19  20-29  30-39  40-49  50-59 | 10.00(0.00)  26.00  16.50 (3.88)  17.24 (4.24)  19.00 (3.42)  19.00 (3.56) |  | 10.25 (0.71)  10.00 (0.00)  13.50 (4.81)  12.40 (5.67)  10.55 (1.29)  12.00 (2.82) |  |
| Age (years)  8-19  20-29  30-39  40-49  50-59 | 13.20 (7.15)  16.50 (3.88)  17.24 (4.24)  19.00 (3.42)  19.00 (3.56) | 0.302 | 10.20 (0.63)  13.50 (4.81)  12.40 (5.67)  10.55 (1.29)  12.00 (2.82) | 0.414 |
| Gender  Male  Female | 16.88 (3.20)  17.35 (5.08) | 0.732 | 11.62 (4.58)  11.71 (3.77) | 0.938 |
| Occupation  Student (Parent)  Unemployed  Retired  Employee | 22.67 (3.05)  16.69 (5.27)  19.00 (3.56)  16.63 (4.01) | 0.129 | 10.14 (0.53)  13.75 (6.29)  13.33 (3.05)  11.30 (3.65) | 0.120 |

P values are obtained from the One-way ANOVA and t-test.

Figure 3: Telephone clinic survey results


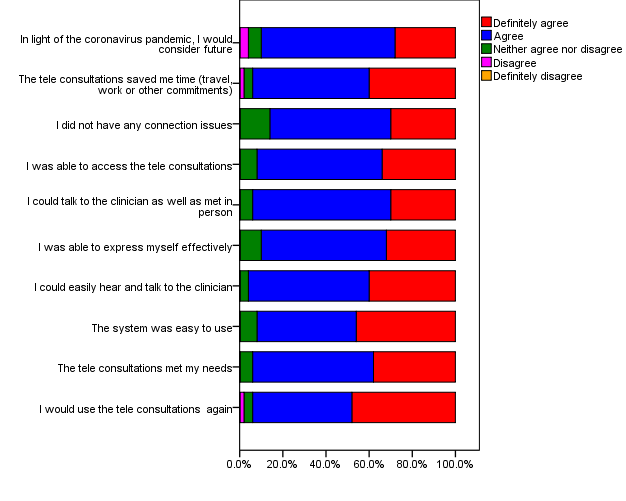


Figure 4: Virtual clinic survey results


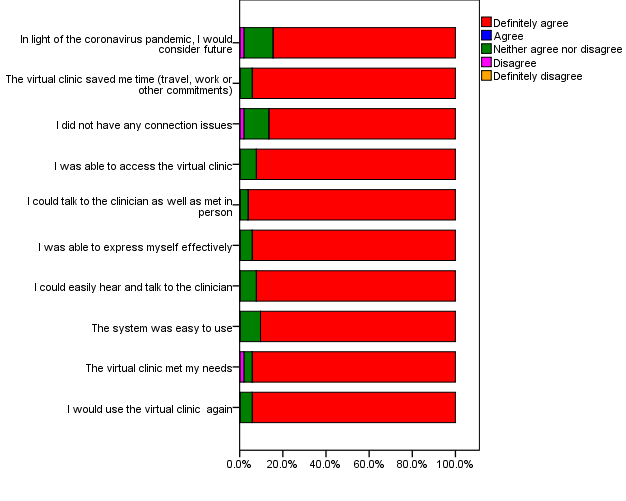


).
